# A fully automated micro‑CT deep learning approach for precision preclinical investigation of lung fibrosis progression and response to therapy

**DOI:** 10.1186/s12931-023-02432-3

**Published:** 2023-05-09

**Authors:** Martina Buccardi, Erica Ferrini, Francesca Pennati, Elena Vincenzi, Roberta Eufrasia Ledda, Andrea Grandi, Davide Buseghin, Gino Villetti, Nicola Sverzellati, Andrea Aliverti, Franco Fabio Stellari

**Affiliations:** 1 Department of Mathematical, Physical and Computer Sciences, University of Parma, Parma, Italy; 2 Department of Veterinary Science, University of Parma, Parma, Italy; 3 Dipartimento di Elettronica, Informazione e Bioingegneria, Politecnico Di Milano, Milan, Italy; 4 Department of Computer Science, Bioengineering, Robotics and Systems Engineering, University of Genoa, Genoa, Italy; 5 Camelot Biomedical System S.R.L, Via Al Ponte Reale 2/20, 16124 Genoa, Italy; 6 Department of Medicine and Surgery, University of Parma, Parma, Italy; 7 Experimental Pharmacology & Translational Science Department, Chiesi Farmaceutici S.P.A, 43122 Parma, Italy

**Keywords:** Bleomycin model, Lung fibrosis, Deep learning, Drug discovery, Micro-computed tomography

## Abstract

Micro-computed tomography (µCT)-based imaging plays a key role in monitoring disease progression and response to candidate drugs in various animal models of human disease, but manual image processing is still highly time-consuming and prone to operator bias. Focusing on an established mouse model of bleomycin (BLM)-induced lung fibrosis we document, here, the ability of a fully automated deep-learning (DL)-based model to improve and speed-up lung segmentation and the precise measurement of morphological and functional biomarkers in both the whole lung and in individual lobes. µCT-DL whose results were overall highly consistent with those of more conventional, especially histological, analyses, allowed to cut down by approximately 45-fold the time required to analyze the entire dataset and to longitudinally follow fibrosis evolution and response to the human-use-approved drug Nintedanib, using both inspiratory and expiratory μCT. Particularly significant advantages of this µCT-DL approach, are: (i) its reduced experimental variability, due to the fact that each animal acts as its own control and the measured, operator bias-free biomarkers can be quantitatively compared across experiments; (ii) its ability to monitor longitudinally the spatial distribution of fibrotic lesions, thus eliminating potential confounding effects associated with the more severe fibrosis observed in the apical region of the left lung and the compensatory effects taking place in the right lung; (iii) the animal sparing afforded by its non-invasive nature and high reliability; and (iv) the fact that it can be integrated into different drug discovery pipelines with a substantial increase in both the speed and robustness of the evaluation of new candidate drugs. The µCT-DL approach thus lends itself as a powerful new tool for the precision preclinical monitoring of BLM-induced lung fibrosis and other disease models as well. Its ease of operation and use of standard imaging instrumentation make it easily transferable to other laboratories and to other experimental settings, including clinical diagnostic applications.

**Supplementary Information:**

The online version contains supplementary material available at 10.1186/s12931-023-02432-3.

## Introduction

Computed tomography (CT) is currently the imaging gold standard for the clinical evaluation of several lung disorders [1–4]. Its miniaturized version, i.e. micro-computed tomography (μCT), represents an invaluable non-invasive tool for investigating the development of lung pathology but also for monitoring the efficacy of new candidate drugs in small-animal models of pulmonary diseases [5, 6].

In all CT applications, including μCT-based preclinical studies, lung segmentation represents the first critical step that precedes the extraction of quantitative CT data such as lung volume, mean lung attenuation (MLA), and related functional parameters [7, 8].

In particular, dynamic lung function biomarkers can only be derived from the segmentation of two reconstructed separate datasets, corresponding to the end-inspiration (P01) and end-expiration (P02) phases [7]. In the absence of such information, only a static view of the intrapulmonary state can be acquired.

Commercially available software allows for semi-automatic lung segmentation. However, in the murine model of pulmonary fibrosis, this software does not correctly delineate severely fibrotic regions, which due to massive collagen deposition and decreased air content, appear as dense as the surrounding tissue. In this situation, manual segmentation is required to correctly segment lung tissue and accurately quantify lung volume as well as MLA. Manual intervention is also necessary to perform additional operations, such as the separation of the whole lung into its left and right portions, which could be important for evaluating disease progression in the two districts, especially in unbalanced/patchy pulmonary diseases that may lead to a different distribution of lesions across the two lobes [9].

Manual segmentation, however, has two main drawbacks that severely hinder its application to large datasets: it prolongs post-processing time by up to 40 min per scan and it is prone to operator-dependent bias [10, 11]. These shortcomings have recently been addressed through the development of artificial intelligence (AI)- and deep learning (DL)-based algorithms for automated lung segmentation in murine models of lung cancer [12] and parenchymal pulmonary diseases [13]. Particularly encouraging results have been reported for DL approaches based on convolutional neural networks (CNN) applied to the segmentations of various organs [14, 15], including lungs [9, 16]. However, to date, none of these tools has been integrated into any drug discovery pipeline.

In the present study, we validated a DL-based approach, previously developed for the automated segmentation of fibrotic lungs [9], in the context of a pharmacological experiment performed in a murine model of pulmonary fibrosis by automatically deriving longitudinal biomarkers from μCT scans. The DL-based model was initially retrained through the incorporation into the original training dataset of additional μCT scans retrieved from a bleomycin (BLM)-induced lung fibrosis model in male mice. This was followed by an evaluation of the extent of fibrosis and its effect on CT parameters both in the whole lung and in separate, left and right, lungs.

A Spearman correlation analysis was then conducted to compare µCT biomarkers and histomorphological endpoints. Also taking advantage of lung lobe-specific inspiratory and expiratory μCT data, this work provides quantitative and more comprehensive information on the responsiveness to Nintedanib (NINT), a human use-approved drug for the treatment of progressive fibrosing interstitial lung diseases [17], of BLM-induced lung fibrotic lesions with different severity.

## Methods

### Ethics statement

The experiment described herein was approved by the intramural animal-welfare committee for animal experimentation of Chiesi Farmaceutici and authorized by the Italian Ministry of Health (protocol number: 809/2020-PR). All procedures were conducted in an AAALAC (Association for Assessment and Accreditation for Laboratory Animal Care) certified facility in compliance with the European Directive 2010/63 UE, Italian D.Lgs 26/2014, the revised “Guide for the Care and Use of Laboratory Animals” [18] and with the Animal Research: Reporting of In Vivo Experiments (ARRIVE) guidelines [19].

### Animals

At first, 23 C57bl/6 male mice, 8 to 10-week-old, provided by Envigo (San Pietro al Natisone, Udine, Italy), were acclimatized to the local vivarium conditions (room temperature: 20–24 °C; relative humidity: 40–70%; 12-h light–dark cycle) for at least 5 days, having free access to standard rodent chow and softened tap water. Nineteen mice were then lightly anesthetized with 2.0% isoflurane delivered in a box and administered with bleomycin (Baxter Oncology GmbH) 10 µg/mouse in 50 μl saline (0.9%), while four mice received only 50 μl saline (hereafter designated as “SAL mice”) via oropharyngeal aspiration (OA) using a micropipette [20]. This procedure was performed on days 0 and 4, as reported in the scheme in Additional file 1: Fig. S1A. The BLM dose utilized in this experiment has been selected to perform routine in-vivo drug screening experiments [20]. On day 7, twelve BLM-treated mice were randomly selected to receive Nintedanib (hereafter called “BLM + NINT mice”) as the reference tool compound since it has been approved for human IPF treatment [21]. All mice (N = 4 SAL, N = 7 BLM, and N = 12 BLM + NINT) were daily treated orally for 2 weeks (from day 7 to day 21), either with the vehicle (1% Tween80 in milliQ water) or Nintedanib (60 mg/kg/day, dissolved in 1% Tween80 in milliQ water) [8, 22]. The Nintedanib dose used in this study was selected based on its established efficacy and previous use in similar experimental models [8, 21, 23]. All appropriate measures were taken to minimize the animals’ pain or discomfort. The pain was evaluated daily through a Visual Analogue Scale (VAS) ranging from 0 (no pain) to 10 (intense pain) by a designated veterinarian or trained technician. Signs of dyspnea, body weight loss ≥ 20, and VAS ≥ 6 were considered as human endpoints (HEP). Animals were also monitored daily and weighed every 2–3 days throughout the experimental procedure (Additional file 1: Fig. S1B). Throughout the experiment reported in the present manuscript, none of the animals reached HEP and no mortality was observed.

### Micro-CT acquisition protocol

Following anesthesia induction and maintenance with 2% isoflurane, mice thoraxes were scanned with a Quantum GX Micro-CT (PerkinElmer, Inc. Waltham, MA) at 7, 14, and 21 days. Images were acquired in free breathing mice with the following parameters: X-ray tube current 88 μA, X-ray tube voltage 90 kV, over a total angle of 360° for a total scan time of 4 min. Each animal was placed in a supine position on the bed of the scanner, and the chest was adjusted to fit within the field of view. A region of interest was positioned over the diaphragm for respiratory gating. The retrospectively gated acquisition protocol was in ‘high speed’ mode, with projections collected in list-mode over a single continuous gantry rotation (each projection is acquired over 16.6 ms). At the end of each acquisition, a window displayed the breathing pattern and the position of the projections that would be used for the reconstruction. During the 4 min of acquisition, about 900 projections (both P01 and P02) are automatically sorted and used for the reconstruction of the two datasets, with the possibility to modify the thresholds to select the more appropriate projections. However, this intervention was not required since our anesthesia protocol [7] was strictly controlled and led to stable breathing rates (100–120 brpm) and uniform time windows for the breathing cycle (500–600 ms) with an average duration of the end-inspiratory/expiratory phases of (32 ± 16) ms and (224 ± 32) ms, respectively. For each acquisition, two stacks of 512 cross-sectional images were automatically reconstructed using a filtered back-projection algorithm with a Ram-Lak filter into two 3D datasets, corresponding to the inspiratory and expiratory breathing phases (i.e. end-inspiration, P01, and end-expiration, P02), with 50 μm isotropic reconstructed voxel size. The CT scanner is calibrated monthly with standard phantoms for noise, uniformity, low contrast, and resolution [24].

### µCT post-processing

The reconstructed datasets were processed by an updated version of the DL-based segmentation model proposed by Vincenzi et al. [9]. An early version of the DL-model, which was trained only on female mice, has been initially retrained using µCT scans from BLM-treated male mice to increase the overall capability of the algorithm to correctly segment female and male lung scans. Details about the model's training and validation are available in the Supplementary Methods in the Additional file 2. In particular, the current model allows the segmentation of the whole lung and of the left and right lobes separately, both in the end-inspiration and end-expiration phases.

From the masks of the whole, left and right lungs (Additional file 2: Supplementary Methods), the algorithm automatically extracted the parameters of interest which are resumed in Table 1. Then, it computed the functional and morphological biomarkers described in Table 2. The aeration compartments were calculated by applying ‘HU preclinical ranges’ [11].

**Table 1:** µCT automatically extracted parameters

µCT readouts				
	Name	Description	Unit	Formula
From P01	${N}_{P01}$	Number of voxels at the end of inspiration phase (P01)	–	Counting of lung voxels in P01
	${V}_{P01}$	Total lung volume at the end of inspiration phase (P01)	mm^3^	${N}_{P01}\cdot \mathrm{voxel size}$
	${MLA}_{P01}$	Mean lung attenuation at the end of inspiration phase (P01)	HU	$\sum_{i=1}^{ {N}_{P01}}{\left(HU\right)}_{i}/{N}_{P01}$
	Air	Volume of air at the end of inspiration phase (P01)	mm^3^	$\frac{{\mathrm{V}}_{\mathrm{P}01}\cdot {\mathrm{MLA}}_{\mathrm{P}01}}{-1000(\mathrm{HU})}$
From P02	${N}_{P02}$	Number of voxels at the end of expiration phase (P02)	–	Counting of lung voxels in P02
	${V}_{P02}$	Total lung volume at the end of expiration phase (P02)	mm^3^	${N}_{P02}\cdot \mathrm{voxel size}$
	${MLA}_{P02}$	Mean lung attenuation at the end of expiration phase (P02)	HU	$\sum_{i=1}^{ {N}_{P02}}{\left(HU\right)}_{i}/{N}_{P02}$
	FRC	Functional residual capacity: Volume of air at the end of inspiration phase (P02)	mm^3^	$\frac{{\mathrm{V}}_{\mathrm{P}02}\cdot {\mathrm{MLA}}_{\mathrm{P}02}}{-1000(\mathrm{HU})}$

**Table 2:** µCT automatically computed biomarkers

µCT biomarkers of interest				
	Name	Description	Unit	Formula
Morphological biomarkers	%Normo	Percentage of parenchyma which is normo-aerated. It reflects the number of no/mild lesions	%	Percent voxels in range [− 860, − 435]*
	%Hypo	Percentage of parenchyma which is hypo-aerated. It reflects the number of moderate lesions	%	Percent voxels in range (− 435, − 121)*
	%Non	Percentage of parenchyma which is non-aerated. It reflects the number of severe lesions	%	Percent voxels in range [− 121, 121]*
	Tissue	Lung volume without gas	mm^3^	${\mathrm{V}}_{\mathrm{P}02}-\mathrm{FRC}$
Functional biomarkers	$\%{Gas}_{P01}$	Percentage of gas volume at the end of inspiration phase (P01)	%	$\mathrm{Air}\cdot 100/{\mathrm{V}}_{\mathrm{P}01}$
	$\%{Gas}_{P02}$	Percentage of gas volume at the end of expiration phase (P02)	%	$FRC\cdot 100/{V}_{P02}$
	Tidal Volume (TV)	Volume of air exchanged between inspiration and expiration	mm^3^	$\mathrm{Air}-\mathrm{FRC}$

Each CT-derived parameter and biomarker was calculated for the whole lung and for separate left and right lungs.

### Assessment of lung fibrosis by histological analysis

Mice were sacrificed on day 21 by anesthetic overdose followed by abdominal aortic bleeding. For histological analysis, the lungs were removed and inflated with a cannula through the trachea by gentle infusion of 0.6 ml of 10% neutral-buffered formalin and fixed for 24 h. Sections of 5 μm were cut with a rotary microtome (Slee Cut 6062; Slee Medical, Mainz, Germany) in dorsal plane and stained with Masson’s trichrome. The whole-slide images were acquired by the NanoZoomer S-60 Digital slide scanner (Hamamatsu). Fibrotic modifications were assessed by the Ashcroft score (AS) scale, by three trained histopathologists in blind [25, 26]. For each sample, several 10X fields were analyzed and morphological changes were graded semi-quantitatively into three classes with different degrees of fibrosis severity: no/mild (from 0 to 3), moderate (equal to 4), and severe (≥ 5) [27]. The average score was calculated, as well as the Ashcroft frequency distribution expressed as percentage of each fibrosis severity class.

### Statistical analysis

A Two-way ANOVA followed by Dunnett’s and Šidák post-hoc test was performed to detect differences between SAL or BLM + NINT mice compared to the BLM group and to evaluate intra-group longitudinal changes in lung CT parameters (day 21 vs. day 7), respectively. The percentage of inhibition (or recovery, according to the parameter) has been calculated for parameters displaying a significant reduction (or augment) in the BLM + NINT group compared to BLM mice at 21 days. For all CT parameters, the paired Student’s t-test was performed to highlight significant differences between the left and right lungs for BLM and BLM + NINT groups at all time-points, and to investigate if Nintedanib treatment displays different anti-fibrotic effects between the two lung lobes. In cases in which normality test failed, non-parametric Wilcoxon test was applied. Two-way ANOVA followed by Dunnett’s t post-hoc test was performed to compare histological outcomes, i.e. average Ashcroft score and Ashcroft frequency distribution, between groups. The paired Student’s t-test was employed to compare the average Ashcroft score and Ashcroft frequency distribution of the right and left lungs. In cases in which normality test failed, non-parametric Wilcoxon test was applied. Finally, the correlation between µCT readouts and the Ashcroft score was assessed by calculating Spearman correlation coefficients. All statistical analyses were performed using Prism 8 software (GraphPad Software Inc., San Diego, California, United States); p < 0.05 was considered statistically significant.

## Results

### DL-based lung segmentation

**Fig. 1:**
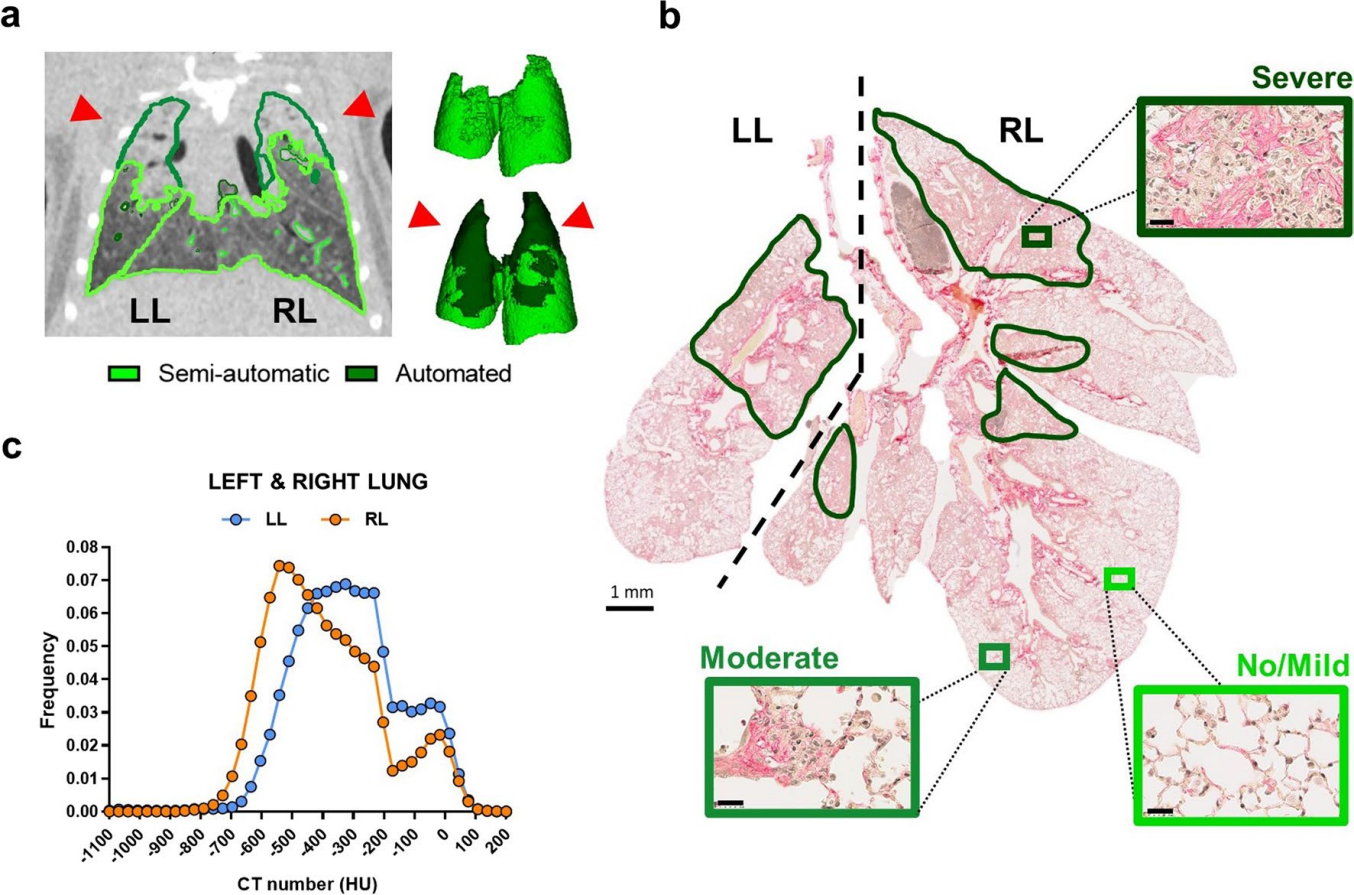
Comparative imaging of a representative lung fibrosis mouse model at 21 days of BLM treatment. **A** A representative 2D coronal µCT slice of a fibrotic lung is shown on the left. The semi-automatic segmentation approach failed to visualize the entire lung (light-green line), while DL-based segmentation allowed the automated segmentation of the whole lung parenchyma (dark-green line). The 3D renderings shown on the right were generated by combining the images obtained with the semi-automatic (light green volume) and the DL-based segmentation approach (dark-green volume). The right and the left lung are indicated as RL and LL, respectively. Red pointed arrows indicate the more severe fibrotic lesions. **B** Histological slice of the lung with a dashed line separating the left and the right lungs (the bar corresponds to 1 mm). Regions of increasingly severe fibrosis are shown as magnified images (bars correspond to 25 µm), boxed by increasingly dark frames outside of the main picture. Fibrosis severity was assessed by Ashcroft score (AS) measurements: no/mild (light green), moderate (green), and severe (dark green). **C** Hounsfield Units (HU) frequency distributions for the left (LL, light blue line) and right (RL, orange line) lungs were determined with the DL-based model

The semi-automatic segmentation approach (*light green edges*) failed to include the severely fibrotic areas, and less aerated portions of the lung, resulting in an incomplete lung volume segmentation (*light-green area*) and an incorrect MLA estimation (Fig. 1A). In contrast, the retrained automated DL-based model succeeded in recognizing the whole lung, detecting even those portions of parenchyma (*dark-green edges* and lung areas in Fig. 1A) that are less or non-aerated due to collagen deposition. As revealed by the overall dataset, the algorithm successfully segmented the entire lung (Additional file 1: Fig. S2), the separate lung lobes (Additional file 1: Fig. S3) as well as normo-, hypo-, and non-aerated lung compartments of untreated and fibrotic mice at all time-points (Additional file 1: Fig. S4). In the representative example shown in Fig. 1A, the more severely fibrotic regions were apical (*red arrows*) and seemingly more extended within the left lobe. Histological analysis performed on the same animal confirmed that the apical regions were indeed characterized by the most severe fibrotic lesions (Fig. 1B, *dark-green areas*), while only mild to moderate lesions were detected in the caudal regions of the lungs. In this case study, the Ashcroft score measurements confirmed a higher abundance of severe fibrotic lesions in the left (average AS = 5.36) compared to the right (average AS = 4.05) lung. Moreover, by separately analyzing the two lung lobes, as allowed by the DL-based approach, we observed that in the left lung the frequency distribution of HU, an indicator of fibrosis, is shifted towards higher HU values compared to the right lung (Fig. 1C). Although the above data derive from a single representative example (but were confirmed and further corroborated by subsequent more in-depth analyses), they illustrate quite clearly the power of our DL-based approach in the dissection and full imaging of the lungs, even under unfavorable, severe fibrosis conditions.

### µCT-DL qualitatively highlights morphological and functional changes in the BLM model of lung fibrosis

**Fig. 2:**
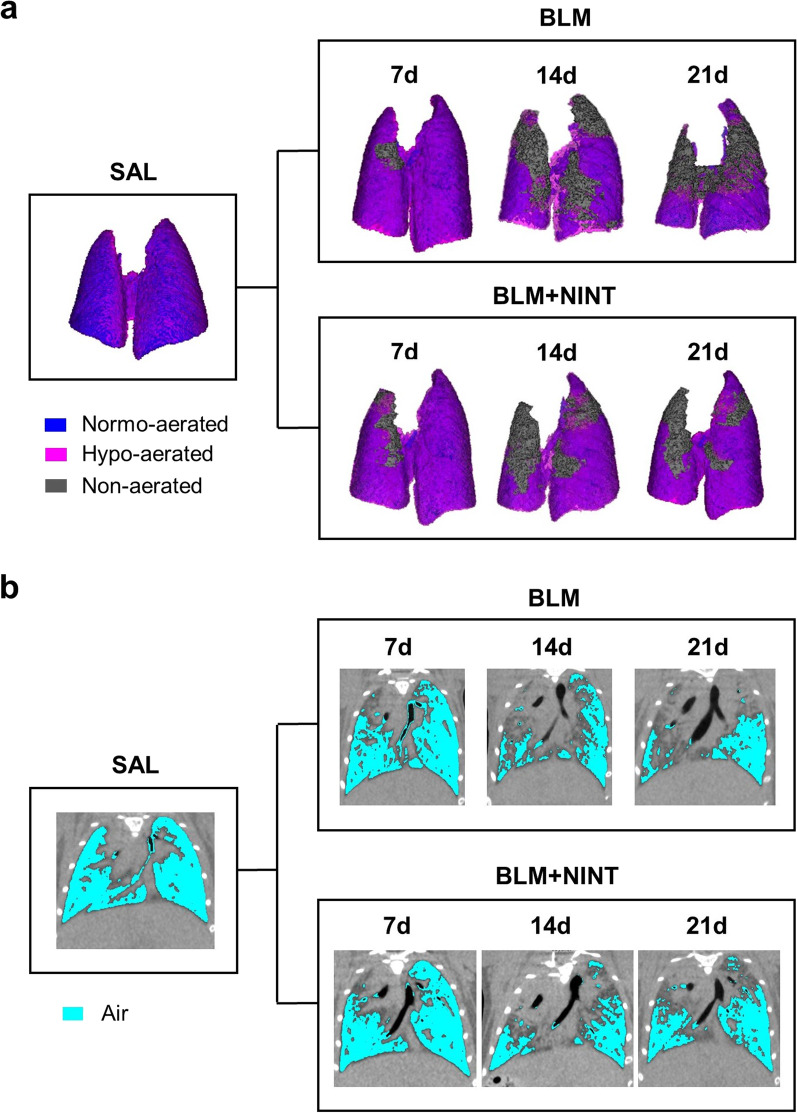
Qualitative longitudinal monitoring of lung fibrosis progression in BLM- and BLM + NINT-treated mice. **A** Representative 3D renderings of the lungs at the end of the end-expiratory phase (P02) derived from a randomly chosen animal per group (SAL, BLM, and BLM + NINT). Different degrees of lung aeration are shown as false colors (blue: normo-aerated; pink: hypo-aerated; grey: non-aerated). 3D renderings of the lungs from BLM- and BLM + NINT-treated mice were generated for each time-point (7, 14, 21 days) in order to longitudinally monitor changes in shape and aeration compartments. The representative SAL lung has 81.4% normo-aerated tissue and 18.6% hypo-aerated tissue. In the BLM and BLM + NINT representative cases the %non-aerated tissue increased from day 7 to 21, respectively from 1.5% to 38.9%, and from 6.2% to 12.2%; the %hypo-aerated compartment increased from day 7 to 21, respectively from 24.7% to 38,4%, and from 36.1% to 39.1%. On the contrary, the %normo-aerated tissue decreased in both groups from day 7 to 21, respectively from 73.8% to 22.7%, and from 57.7% to 48.7%. **B** Representative µCT 2D coronal slices of the same lung images shown in **A** acquired at the end-expiratory phase (P02); cyan-colored, low-intensity pixels represent the air content of the lungs

Representative 3D renderings of SAL, BLM, and BLM + NINT mice at different time-points and the corresponding aeration compartments (normo-, hypo-, and non-aerated) in P02 are shown in Fig. 2A. As expected, the SAL control displayed a smaller left lung compared to the right one. About 80% of the total lung was classified as normo-aerated, whereas the remaining tissue was considered as hypo-aerated including a small portion detected at the boundaries due to respiratory motion [11, 28]. BLM administration caused an acute pulmonary inflammation on day 7, resulting in a marked increase in the total lung. From day 7 to day 21, fibrosis progressed in the BLM-treated mice resulting in an increase of the non-aerated compartment and in a change of shape in the apical lung regions. Overall, similar features were detected in the BLM and in the BLM + NINT group on day 7, whereas a smaller fraction of non-aerated tissue was observed in the BLM + NINT mice on day 21.

The air content decreased dramatically from day 7 to day 21 in the BLM-treated mice compared to the SAL controls (Fig. 2B), but the caudal portions of the right lungs remained well-aerated. A strong decrease in lung aeration was also observed in the BLM + NINT mice. This was particularly evident during the first week of treatment (day 7 to day 14) but was markedly attenuated on day 21. Even if the aforementioned instances showcase a representative animal from each group, they effectively demonstrate how the subsequent quantifications, allowed by the µCT-DL analysis, will accurately depict actual longitudinal morphological and functional alterations in the lungs.

### Longitudinal progression of lung morphological biomarkers assessed by µCT-DL

Results obtained for the SAL control group (reported in Additional file 1: Fig. S5) showed that each metric was stable over time, again with a significant difference between the left and the right lungs (Additional file 1: Fig. S5C–E and Additional file 1: Table S1). Based on these SAL data, all µCT parameters of the BLM- and BLM + NINT-treated groups were normalized with respect to the mean value of each parameter measured in the control group, except for %Non which is not present in healthy mice (equal to 0).

**Fig. 3:**
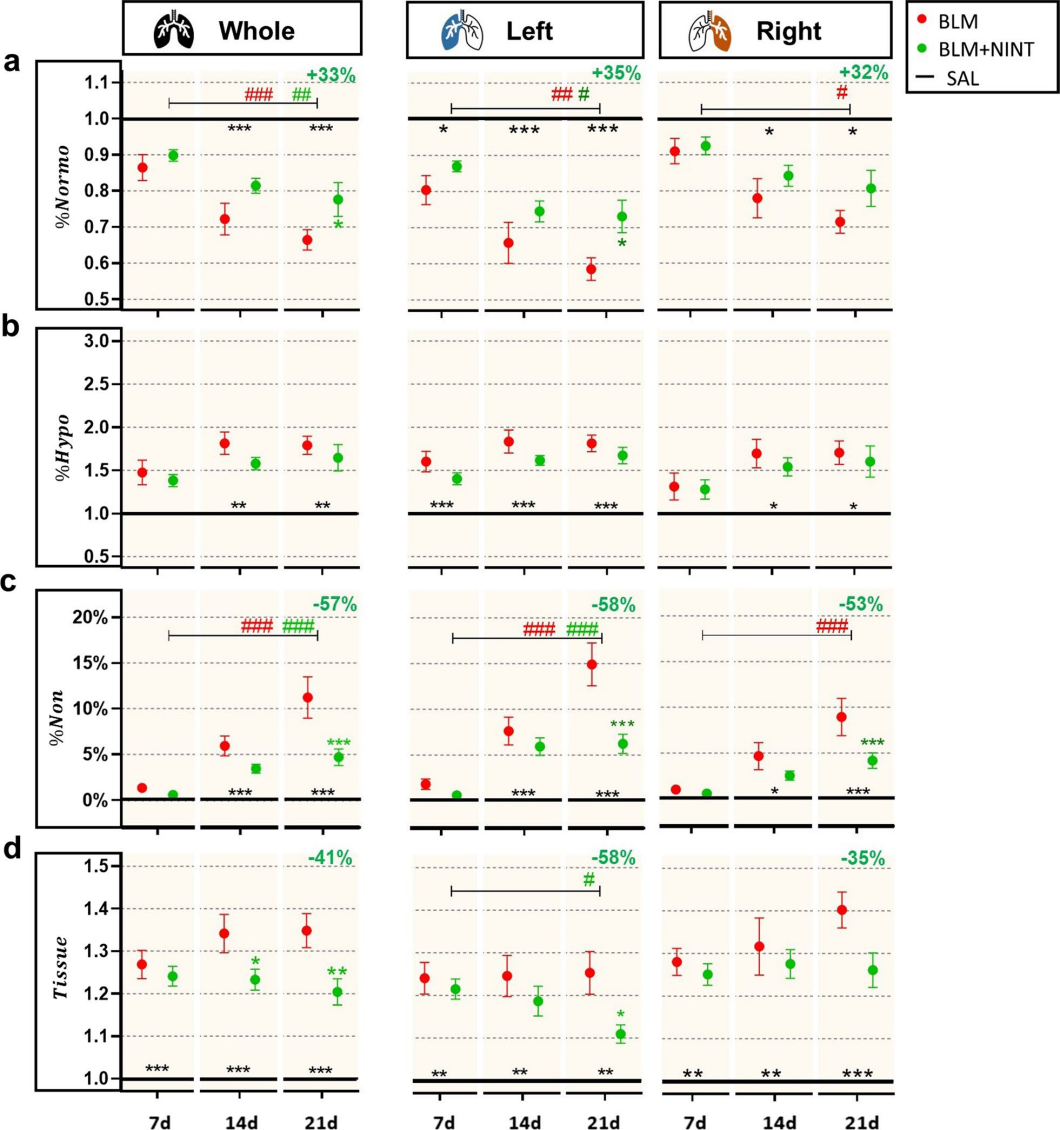
Longitudinal assessment of morphological µCT biomarkers in the P02 phase for the whole, left, and right lungs. **A** Quantification of the Normo-aerated compartment expressed as percentage (%Normo). **B** Quantification of the Hypo-aerated compartment expressed as percentage (%Hypo). **C** Quantification of the Non-aerated compartment expressed as a percentage (%Non). **D** Lung volume without gas (Tissue) quantification. All values reported for the BLM and BLM + NINT groups were normalized with respect to the mean values of the SAL group averaged on days 7, 14, and 21, except for %Non which is expressed as absolute percentage value. The black lines set at 1.0 represent the “untreated” condition obtained by dividing the SAL mean value by itself, while in **C** the black line is set at 0. BLM (red) and BLM + NINT (green) data are given as mean ± SEM. Statistical significance of longitudinal changes of CT parameters in the BLM and BLM + NINT groups was assessed by Two-way ANOVA followed by Šidák post-hoc test (^#^p < 0.05; ^##^p < 0.01; ### p < 0.001). Statistical significance of differences between groups was calculated by Two-way ANOVA followed by Dunnett’s t post-hoc test (*p < 0.05; **p < 0.01; ***p < 0.001 vs. BLM group) and the relative percentage of inhibition (−) or recovery (+) at 21 days was reported at the top right-side of each plot

The normo-aerated volume (%Normo) was significantly lower in BLM and BLM + NINT mice compared to the SAL group at days 14 and 21 in the whole and right lungs, and at all time-points in the left lungs (Fig. 3A). From day 7 to day 21, %Normo for the whole lungs significantly decreased in both the BLM and BLM + NINT groups but with different rates (p < 0.001 and p < 0.01, respectively). As a result, on day 21 a significant recovery of %Normo in the BLM + NINT group compared to the vehicle was measured (+ 33%, p < 0.05). Although less pronounced, a similar trend was observed in the left lung where the decline in %Normo was significant from day 7 to day 21 both in the BLM and BLM + NINT groups (p < 0.01 and p < 0.05, respectively), but significantly slowed down by Nintedanib treatment compared to BLM group at day 21 (+ 35%, p < 0.05). In the right lobes, a significant longitudinal decrease of %Normo was apparent only in BLM mice (p < 0.05).

The percentage of hypo-aerated volume (%Hypo) was significantly increased in BLM and BLM + NINT mice compared to the SAL controls at all time-points, except for the whole and right lungs on day 7 (Fig. 3B). However, despite slight fluctuations, %Hypo remained stable over time in both groups.

The non-aerated compartment (%Non) markedly progressed from day 7 to day 21 in both the BLM and the BLM + NINT groups in the whole lungs (p < 0.001) and in the left lungs (p < 0.001) (Fig. 3C). In the right lungs, a longitudinal increase of %Non reached significance only in the BLM group (p < 0.001). Moreover, as revealed by a comparison between BLM and the BLM + NINT-treated mice, a significant reduction of the fractional non-aerated compartment by Nintedanib treatment could be detected on day 21 both in the whole lung (− 57%, p < 0.001 vs. BLM) and in individual lung lobes (− 58% and − 53%, p < 0.001 vs. BLM, in the left and right lung, respectively).

The non-gas volume of the lung (Tissue), whose variations are due to tissue edema caused by inflammation and to collagen deposition*,* increased in both the BLM and the BLM + NINT groups compared to the SAL control (Fig. 3D). Nintedanib treatment significantly reduced the tissue component of the whole and left lung parenchyma at day 21 (p < 0.01 and p < 0.05, respectively).

Significant differences in morphological parameters, and thus in the severity of fibrotic lesions, between the right and the left lungs are evidenced in Table 3. Indeed, on days 14 and 21, the decline of the normo-aerated compartment, along with the accumulation of non-aerated regions, resulted to be unequal between the two lobes, in agreement with the hypothesis that fibrosis progression preferentially occurs in the left lobe. On day 7, no significant difference in the Tissue component was detected between the two lung lobes both in the BLM and in the BLM + NINT group. However, at later time-points, Tissue in the right lungs was higher compared to the left lung in both groups, thus indicating an inflammatory component that tends to last longer in the former district.

**Table 3:** p-values derived from a paired Student’s t-test analysis comparing the morphological biomarkers measured in the right and left lungs both in the BLM and BLM + NINT groups at each time-point

Paired t-test: left vs. right lungs						
Morphological biomarkers	Day 7		Day 14		Day 21	
	BLM	BLM + NINT	BLM	BLM + NINT	BLM	BLM + NINT
%Normo	**	***	*	**	**	**
%Hypo	*	ns	ns	ns	ns	ns
%Non	ns	ns	*	***	**	***
Tissue	ns	ns	*	**	**	***

ns p > 0.05; *p < 0.05; **p < 0.01; ***p < 0.001

### Longitudinal progression of functional µCT biomarkers

Total expiratory lung volume (V_P02_) was significantly increased on day 7 in the BLM and BLM + NINT groups compared to the SAL control, both in the whole parenchyma and in individual lung lobes, suggesting that this parameter may reflect the inflammatory response to BLM administration, as previously reported [20, 23]. V_P02_ tended to decrease, although non-significantly in the BLM group, at subsequent time-points in the whole and in the left lungs, suggesting that on day 21 the inflammation in the left lung was replaced by fibrotic and scarred tissue. On the contrary, only slight fluctuations could be observed in the right lungs, also suggesting a compensatory mechanism. Consequently, the effect of Nintedanib slowing collagen deposition resulted in a longitudinal drop of V_P02_ only in the left lobe (p < 0.05).

**Fig. 4:**
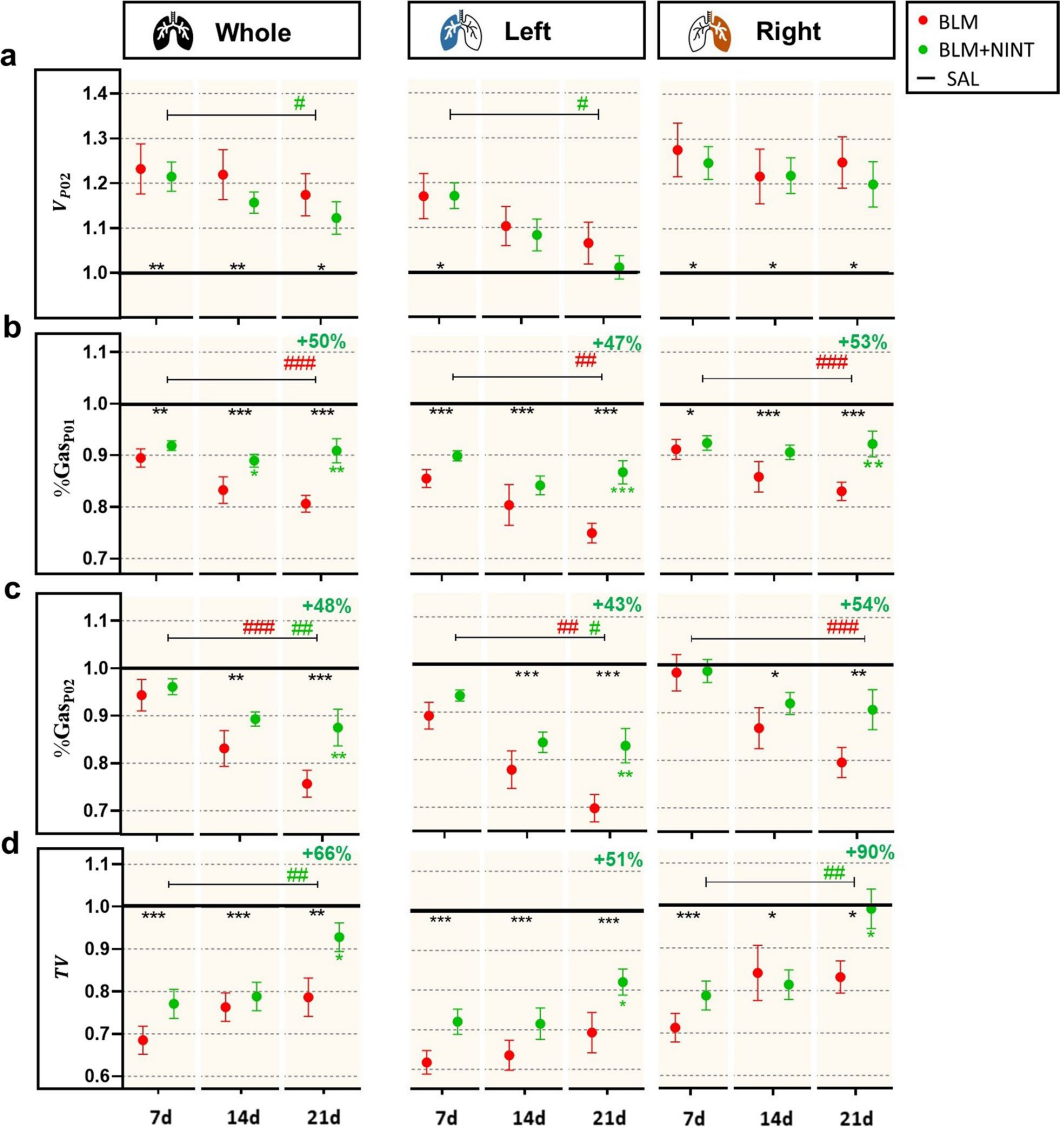
Longitudinal assessment of functional µCT biomarkers for the whole, left and right lungs in the respiratory phases P02 and P01. **A** Quantification of the Total Lung Volume at the end of expiration (V_PO2_). **B** Quantification of the air content at the end of inspiration expressed as percentage (%Gas_P01_). **C** Quantification of the air content at the end of expiration expressed as percentage (%Gas_P02_). **D** Quantification of the Tidal Volume (TV). All values reported for the BLM and BLM + NINT groups were normalized with respect to the mean values of the SAL group averaged on days 7, 14, and 21. The black lines arbitrarily set at 1.0 represent the “untreated” condition obtained by dividing the SAL mean value by itself. BLM (red) and BLM + NINT (green) data are given as mean ± SEM. Statistical significance of the longitudinal changes of CT parameters in the BLM and BLM + NINT groups was assessed by Two-way ANOVA followed by Šidák post-hoc test (^#^p < 0.05; ^##^p < 0.01; ^###^p < 0.001). Statistical significance of differences between groups was determined by Two-way ANOVA followed by Dunnett’s t post-hoc test (*p < 0.05; **p < 0.01; ***p < 0.001 vs. BLM group) and the relative percentage of inhibition (−) or recovery (+) at 21 days was reported at the top right-side of each plot

The percentage of air content (%Gas_P01_) at the end-inspiratory phase declined in both BLM and BLM + NINT-treated groups compared to SAL at all time-points (Fig. 4B). A significant drop in %Gas_P01_ was observed in the whole lung parenchyma as well as in the left and right lungs of BLM mice from day 7 to day 21 (p < 0.001, p < 0.01, and p < 0.001, respectively). Nintedanib treatment stabilized the %Gas_P01_ decline, which was (on average) + 50% higher compared to the BLM group in the whole lungs (p < 0.01), + 47% in the left lungs (p < 0.001), and + 53% in the right lungs (p < 0.01). %Gas_P01_ in the whole lungs of BLM + NINT-treated mice was also significantly higher than in the BLM group on day 14 (p < 0.05).

%Gas_P02_ significantly decreased from day 7 to day 21 in both the BLM and the BLM + NINT groups at the whole lung level (p < 0.001 and p < 0.01, respectively) and in the left lung lobes (p < 0.01, p < 0.05, respectively) (Fig. 4C). In the right lungs, a marked decline of %Gas_P02_ was found only in BLM mice (p < 0.001). On day 21, a significant recovery of %Gas_P02_ was measured in BLM + NINT mice compared to the BLM group both in the whole and in the left lungs (p < 0.01), thus pointing to a left lobe-preferential effect of Nintedanib.

In BLM mice, the Tidal Volume (TV) of the whole, right, and left lungs resulted significantly impaired compared to the SAL controls at all time-points (Fig. 4D). The single-lobe analysis performed on BLM mice revealed that in the left lungs, this impairment was constant over time (p < 0.001 vs. SAL) while it attenuated from day 7 (p < 0.001) to day 21 (p < 0.05) in the right lungs; however, no significant longitudinal differences were detected. In the whole lung and in the right lung lobes, the Nintedanib treatment resulted in a significant increase of the TV from day 7 to day 21 (p < 0.01) and was significantly higher compared to the TV in the BLM group at the end of the treatment (p < 0.05). Comparing data from the right and the left lungs (Table 4), the increment in V_P02_ resulted weaker in the left lungs at all time-points in both the BLM and the BLM + NINT groups, suggesting a compensatory effect of the right lobes and indicating a stronger inflammatory response to BLM than their contralateral. Similarly, %Gas_P02_ values in the left lungs were statistically lower compared to those measured in the right lungs at all time-points both in the BLM and in the BLM + NINT group. These differences were less marked, but still present, at the end-inspiratory phase, indeed %Gas_P01_ values tended to be higher in the right lobes in both BLM and BLM + NINT groups. No significant differences in TV between the left and the right lungs could be detected on days 7 and 14. On day 21, however, TV values in the right lungs were statistically higher than those of the left lungs in both BLM and BLM + NINT groups, in agreement with the hypothesis of a compensatory effect of the right lung.

**Table 4:** p-values derived from a paired Student’s t-test analysis comparing the functional biomarkers measured in the right and left lungs both in the BLM and BLM + NINT groups at each time-point

Paired t-test: left and right lobes						
Functional biomarkers	Day 7		Day 14		Day 21	
	BLM	BLM + NINT	BLM	BLM + NINT	BLM	BLM + NINT
${V}_{P02}$	***	***	*	**	*	**
%Gas_P01_	*	ns	*	ns	ns	*
${\%Gas}_{P02}$	*	*	*	**	*	**
TV	ns	ns	ns	ns	*	*

ns p > 0.05; *p < 0.05; **p < 0.01; ***p < 0.001

### Histological assessment of lung fibrosis and the anti-fibrotic effect of Nintedanib

**Fig. 5:**
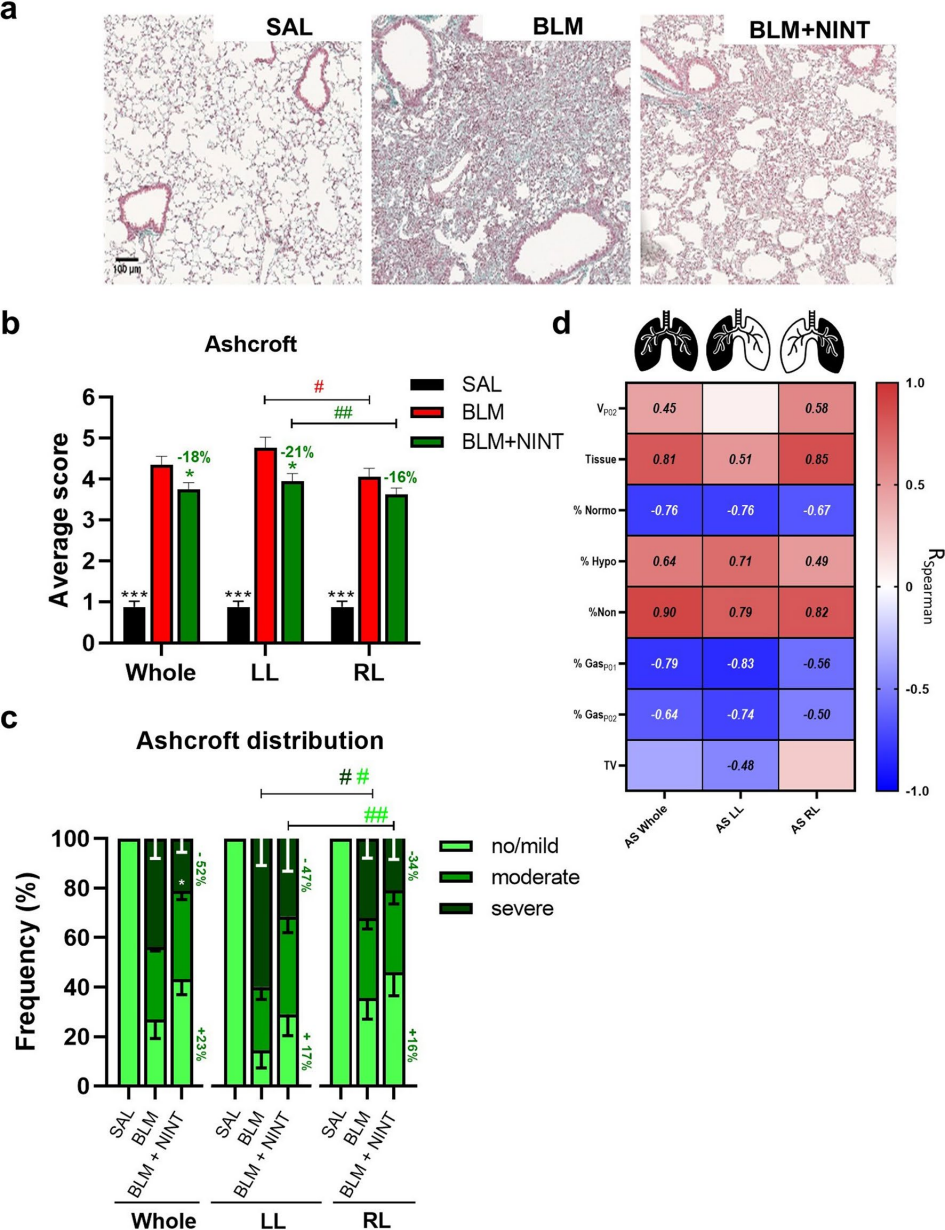
Histological assessment of fibrosis progression in BLM model and Nintedanib effect. **A** Representative images of Masson’s trichrome stained histological sections from SAL (left), BLM (middle), and BLM + NINT (right) mice at 21 days. **B** Ashcroft score (AS) quantification for the SAL, BLM, and BLM + NINT groups in the whole, left (LL), and right (RL) lungs as indicated. Statistical significance of the differences between groups for the whole lung were calculated by Two-way ANOVA followed by Dunnett’s t post-hoc test (*p < 0.05; ***p < 0.001 vs. BLM group), whereas a Student’s t-test analysis was used to evaluate the statistical significance of AS differences between the left and the right lobe of the same group (^#^p < 0.05; ^##^p < 0.01). **C** AS class frequency distribution (no/mild, moderate, and severe) for the whole, left, and right lungs. Statistical differences between the class frequencies of BLM and BLM + NINT groups were evaluated via Two -way ANOVA followed by Dunnett’s t post-hoc test (*p < 0.05). Student’s t-test analysis was used to evaluate the statistical significance of AS class frequency differences between the left and the right lobe of the same groups (^#^p < 0.05; ^##^p < 0.01). **D** Heat-map representation of Spearman correlation coefficients (R) between CT-derived parameters and AS in the whole, left, and right lungs; no significant correlations (p-value < 0.05) have not been reported

The SAL controls displayed an overall normal lung architecture with no detectable alterations, whereas BLM mice lungs were characterized by evident fibrotic lesions with confluent conglomerates of substitutive collagen and inflammatory infiltrates, as reported in Fig. 5A, that appeared less extensive in the BLM + NINT group.

As expected, the mean AS significantly increased in the BLM group compared to the SAL controls, both in the whole lung parenchyma and in individual lung lobes (p < 0.001, Fig. 5B). Nintedanib treatment significantly reduced the AS in the whole parenchyma and in the left lung (− 18% and − 21% respectively; p < 0.05 vs. BLM), whereas no significant difference between the BLM and BLM + NINT groups was observed in the right lung. In keeping with µCT data, histological assessment of fibrosis confirmed that the left lungs of both BLM and BLM + NINT groups were significantly more affected compared to their respective right lungs (p < 0.05 and p < 0.01, respectively).

Predictably, the lungs of the SAL group were totally classified as tissue with no/mild lesions, both at the whole lung and individual lobe levels (Fig. 5C). Moderate and severe fibrotic lesions were prominent, instead, in BLM and BLM + NINT lungs. Overall, Nintedanib inhibited lung fibrosis compared to the BLM group, especially by reducing the frequency of severe lesions (severe: − 52%, p < 0.05 vs. BLM group) and partially recovering no/mild lesions percentage (+ 23%). Also in this case, when the two lung lobes were separately examined, a greater frequency of severe fibrotic areas was observed in the left lungs compared to the right lungs of the BLM group (p < 0.05), and interestingly, Nintedanib strongly reduced more severe fibrosis especially in left lungs (severe: − 47%, p < 0.05 vs BLM) rather than in right lungs (severe: − 34%).

All µCT-derived biomarkers measured at day 21 in the SAL, BLM, and BLM + NINT groups were compared to the AS assessed in the whole lung parenchyma and in the left and right lungs (Fig. 5D). As expected, only the volumetric biomarkers (values not underlined in Fig. 5D) were found not to be correlated with AS (p > 0.05).

Despite the bidimensional nature of histological measurements, a good correlation with µCT biomarkers was observed in the whole lung and in the left lobe. This was markedly reduced in the case of the right lobes, likely due to the difficulty of precisely determining the AS in the four right lobes through a single cut of the entire lung. Nevertheless, µCT-derived parameters more closely related to the extent of fibrosis (non-aerated volume and tissue lung volume) were found to be well-correlated with the Ashcroft score both in the left and in the right lung.

## Discussion

Effective preclinical discovery of new drugs requires the development of reliable animal models of human diseases and highly efficient approaches for their detailed investigation. In various animal models, µCT imaging has proven to be a powerful tool to visualize and precisely quantify the dynamic evolution and regional severity of disease [20, 23]. However, to take full advantage of this imaging approach, it is necessary to automate the post-processing phase of analysis, including lung segmentation. This can be particularly challenging in the case of degenerative pathologies such as lung fibrosis, where fibrotic lesions are often only partially detected by commercial software requiring highly time-consuming and operator-bias-prone manual interventions [9]. Applied to an established mouse model of BLM-induced lung fibrosis, this work documents, for the first time, the ability of a retrained fully automated DL-based algorithm to improve and speed up lung segmentation and the measurement of morphological and functional biomarkers in both the whole lung parenchyma and in individual lung lobes from both inspiratory and expiratory μCTs. Targeted validation was achieved by launching the DL-based algorithm against the entire µCT images dataset of a pharmacological experiment containing 46 scans per time-point. The time required for segmentation and biomarker information extraction was cut down from 40 to 10 min per image, thus reducing the cumulative time required to analyze the entire dataset from 45 days to one day. The DL-guided analysis enabled the automatic extrapolation of µCT-derived morphological and functional biomarkers. This allowed us to longitudinally investigate fibrosis progression and response to Nintedanib, which has recently been approved for progressive pulmonary fibrosis in humans [29, 30] using both inspiratory and expiratory μCTs.

We paid special attention to the separate examination of the left and the right lungs, which is simply unfeasible with manual segmentation, because we suspected a marked difference in the intensity of the pro-fibrotic effect induced by BLM in the two lung lobes, caused by their anatomical and size differences, with the left lobe covering approximately only one-third of the total lung volume [31]. Due to this size difference, following an initially balanced distribution of BLM (delivered by oropharyngeal aspiration) at the tracheal bifurcation between the left and the right bronchus, the local BLM concentration becomes significantly higher in the left lung, which thus tends to develop more severe and progressive fibrotic lesions. The potentially confounding effects associated with this uneven BLM accumulation were successfully addressed by our µCT DL analysis, which allowed an accurate evaluation of the spatial distribution of fibrotic lesions.

Multiple lung biomarkers, both morphological and functional, were longitudinally assessed in parallel and µCT-derived data were systematically compared with those obtained from more conventional, especially histological, analyses. By investigating morphological biomarkers at the whole-lung level, we found their progression to be consistent with the BLM-induced model of lung fibrosis [20, 23]. A better understanding of the model and its progression was achieved through the separate analysis of the right and the left lungs. In both BLM- and BLM + NINT-treated animals, the left lung was characterized by smaller normo-aerated areas and larger fibrotic (non-aerated volume) regions compared to the right lung. These data, together with the results of histological analyses conducted in parallel, further corroborate the notion that the left lung is significantly more affected than the right lung by BLM administration. Despite the limited number of animals employed in this study, the overall consistent results we obtained by automated µCT DL and histological analyses strongly suggest that our approach can reliably guide anti-fibrotic therapy evaluation in-vivo. Specifically, the automated µCT-DL algorithm allowed for accurate segmentation of the lungs and extraction of quantitative parameters, which were consistent with the findings from the histological analysis. Another important practical correlate of the reliability and non-invasive nature of µCT-DL consists in a reduction of the number of animals required per experiment, in agreement with the 3R goals for animal protection (Refinement, Replacement, Reduction) and its ongoing reinforcement [29]. It should be noted, however, that while micro-CT protocols involving weekly follow-up are not associated with radiotoxicity effects [32] and we did not detect the occurrence of any histopathological changes following irradiation on the SAL group by standard histological analysis, previous studies have suggested that MRI could serve as a non-ionizing radiation imaging alternative for quantifying lung injury and evaluating the effects of pharmacological treatments in the bleomycin murine model [33, 34].

In both lungs, Nintedanib significantly ameliorated various morphological and functional parameters. From the longitudinal analysis of the morphological biomarkers, we found that Nintedanib slowed the progression of those parameters that are causally related to the most severe and progressive fibrotic areas (e.g., non-aerated volume and tissue); thus, Nintedanib slowed collagen deposition, leading to the pharmacological effect to be particularly evident in the left lungs where bleomycin-induced fibrotic lesions are more severe and progressive, also suggesting that harsher lesions might be more responsive to the pharmacological treatment as reported in clinical studies [27].

Among functional biomarkers, total lung volume turned out to be the best indicator of the acute, BLM-induced inflammatory phase associated with the fluid accumulation and edema detected on day 7. This early inflammation is a typical feature of the BLM-induced fibrosis mouse model and tends to diminish at later time-points [20]. Similarly, ${\mathrm{V}}_{\mathrm{P}02}$ increased at both the whole and individual lung levels on day 7 and declined thereafter in the whole and left lungs, on the contrary, the right lungs remained stable over time, suggesting that inflammation vanishes faster in the left lobes.

This observation together with disease progression highlighted by longitudinal functional biomarkers evaluation suggested a marked compensatory mechanism of the right lobe.

Indeed, at all time-points, higher %Gas_P02_ and TV values appeared to be concentrated in the right lung of BLM- and BLM + NINT-treated mice. This compensatory response, which has never been described before, could lead to an overestimate of the effects of anti-fibrotic drug candidates on ventilatory and mechanical lung parameters, such as forced vital capacity and overall functionality of the respiratory system, if they are not determined longitudinally. We can hypothesize, that compensatory effects occurred in the caudal part of the right lobes since the histological analysis unveiled that severe fibrotic lesions are mainly located in lung apical regions.

The possibility afforded by µCT-DL of automatically and non-invasively deriving in-vivo longitudinal biomarkers associated with different sites and stages of fibrosis development offers several advantages. First, since each mouse acts as its own control, experimental variability is significantly reduced. Second, the measured biomarkers can be quantitatively compared across experiments, increasing the overall robustness of in-vivo acquired data. Finally, since the post-processing stage is fully automated and free of any operator-dependent bias (even though segmentation and data acquisition are supervised), the ensuing results are more consistent and reproducible.

The above improvements compared to more conventional bioanalytical approaches are expected to advance explorative in-vivo drug discovery studies, especially regarding a more effective (i.e., earlier and more reliable) identification of the most promising drug candidates, ultimately leading to a significant cutback of the overall cost of drug discovery [35].

The present study has some limitations. First, our deep-learning model requires access to a computing cloud, which may not be affordable for all researchers. Additionally, while the algorithm accurately segments the lung lobes and excludes major blood vessels, minor vessels may be still included in the segmented lungs. Also, a limited number of individuals per group was included in the study. Nevertheless, significant differences were still found between groups suggesting the sensitivity of the proposed method. Despite these limitations, our work provides valuable insights into the application of deep-learning models in preclinical pharmacological experiments for the study of pulmonary fibrosis and the response to putative treatments, which can help guide future research in this field.

Full validation of our µCT-DL approach as a routine, general-use bioanalytical tool will have to await its utilization by other preclinical investigation laboratories. However, its ease of operation and the fact that it entirely relies on standard imaging instrumentation, makes us quite confident about its successful transfer to other laboratories and to other preclinical settings in addition to pulmonary research, perhaps including clinical diagnostic applications, which is recently moving towards the identification of radiological markers of disease progression for both diagnosis and prognosis purposes [2, 36, 37].

Finally, also worthy of note is the clinical translation potential, exemplified here for Nintedanib, of the mode of action information on established or new candidate drugs that can be acquired with the precision in-vivo preclinical studies afforded by artificial intelligence-guided imaging.

## Supplementary Information

**Additional file 1: Figure S1.** Schematic representation of the experimental setting and body weight variation. **Figure S2.** Qualitative evaluation of the CT DL model performance. **Figure S3.** CT numbers frequency distribution in the left and right lobes. **Figure S4.** Validation of the DL-based model for the automated segmentation. **Figure S5.** Longitudinal assessment of some derived CT parameters measured from the whole lung and for separate left and right lungs in Saline mice. **Table S1.** p-values derived from a paired Student’s t-test analysis comparing the biomarkers measured in the right and left lungs of SAL animals at each time-point.

**Additional file 2: Table S2.** Data partitioning by time point, acquisition phase, and disease prevalence. **Table S3.** A summary of dice score main statistics in multi-view aggregation stage.

## Data Availability

All data generated or analyzed during this study are included in this published article and its supplementary information files.

## Abbreviations

CT: Computed tomography
µCT: Micro-computed tomography
MLA: Mean lung attenuation
AI: Artificial intelligence
DL: Deep learning
CNN: Convolutional neural networks
NINT: Nintedanib
BLM: Bleomycin
OA: Oropharyngeal aspiration
SAL: Saline
VAS: Visual Analogue Scale
HEP: Humane Endpoint
HU: Hounsfield Units
FRC: Functional residual capacity
AS: Ashcroft score
