# Supplementary material for: A fully automated micro‑CT deep learning approach for precision preclinical investigation of lung fibrosis progression and response to therapy
Source: Respir Res. 2023 May 9;24:126. doi: 10.1186/s12931-023-02432-3 (PMC10170869; doi:10.1186/s12931-023-02432-3)
Supplement: Supplementary file 1 — Additional file 1: Figure S1. Schematic representation of the experimental setting and body weight variation. Figure S2. Qualitative evaluation of the CT DL model performance. Figure S3. CT numbers frequency distribution in the left and right lobes. Figure S4. Validation of the DL-based model for the automated segmentation. Figure S5. Longitudinal assessment of some derived CT parameters measured from the whole lung and for separate left and right lungs in Saline mice. Table S1. p-values derived from a paired Student’s t-test analysis comparing the biomarkers measured in the right and left lungs of SAL animals at each time-point. [file 12931_2023_2432_MOESM1_ESM.docx]

**Supplementary Figures and Tables**

**Figure S1**

**A**


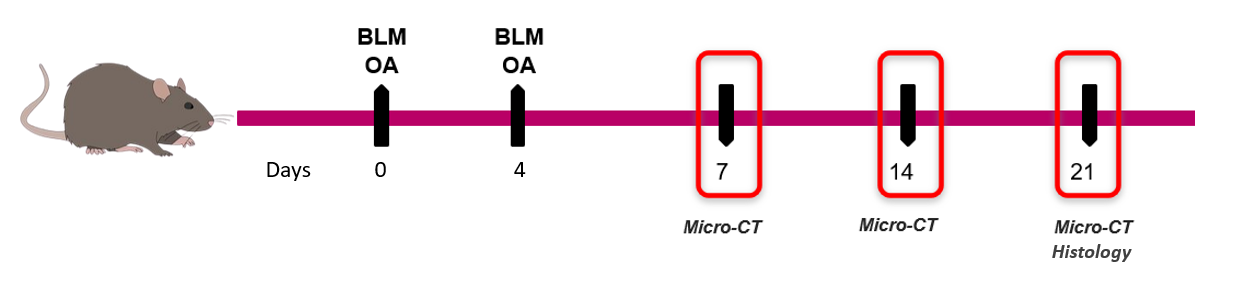


**B**


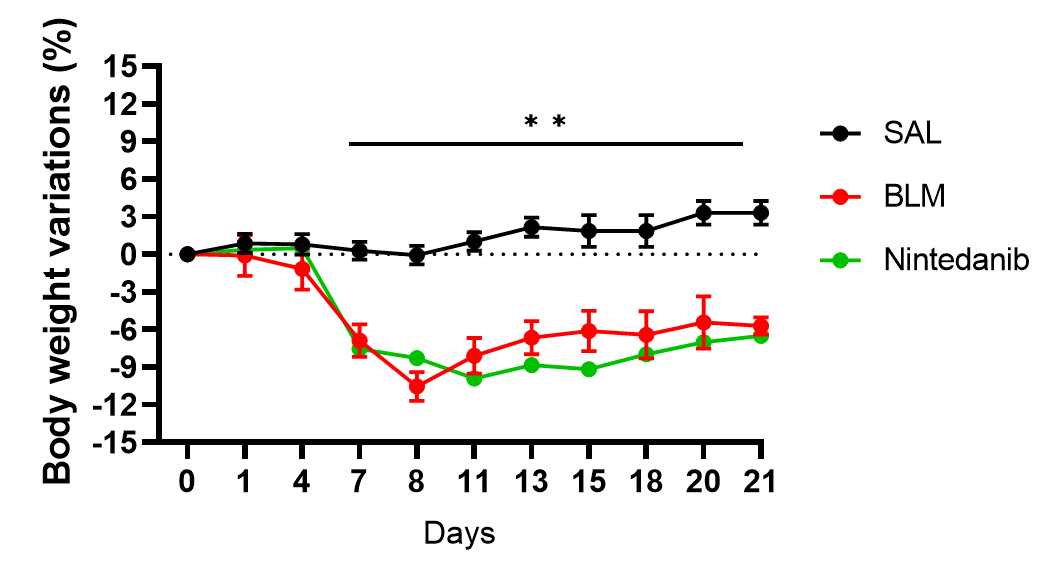


**Figure S1. Schematic representation of the experimental setting and body weight variation.**

(A) Twenty-three C57Bl/6 male mice were treated on day 0 and 4 either with saline (N=4) or BLM (10 μg each, N=19) by oropharyngeal administration. On day 7, BLM-treated mice were randomized to be treated with Nintedanib (N=12) or vehicle (N=7). Micro-CT (μ-CT) was longitudinally performed on days 7, 14, and 21. All animals were euthanized on day 21 to collect lungs for histological assessment of lung fibrosis. (B) Body weight variations were reported for each group as a percentage compared to the baseline (day 0) values. Data were shown as mean ± SEM. Statistical analysis was performed via two-way ANOVA followed by Dunnett’s test for multiple comparisons: ** p < 0.01 vs. saline group.

**Figure S2**


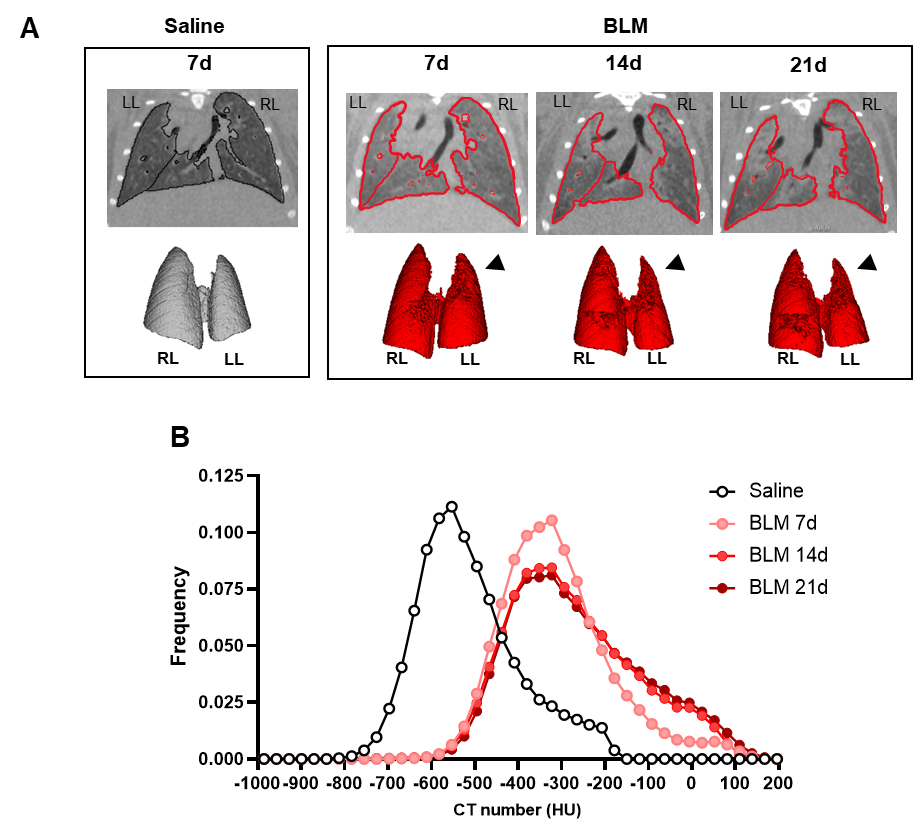


**B**

**A**

**SAL BLM**

**Figure S2. Qualitative evaluation of the** $\boldsymbol{\mu}$**CT DL model performance**

(A) On the left, $\mu$CT 2D coronal slices of a saline mouse acquired at the end of expiration (P02), and the respective 3D rendering obtained by DL segmentation model. The right lung and the left lung are respectively indicated as RL and LL. On the right, $\mu$CT 2D coronal slices of a representative BLM mouse acquired at the end of expiration (P02) at all time points, and the respective 3D renderings. The right lung and the left lung are respectively indicated as RL and LL. Black pointed arrows indicate the more severe fibrotic lesions. (B) HU frequency distributions of the automatically segmented CTs of the same lungs of (A).

**Figure S3**

**B**

**A**

**C**

**Figure S3. CT numbers frequency distribution in the left and right lobes**

CT numbers mean frequency distributions of the right (orange) and left (blue) lungs on day 21 evaluated in SAL (A), BLM+NINT (B), and in BLM (C) groups.

**Figure S4**


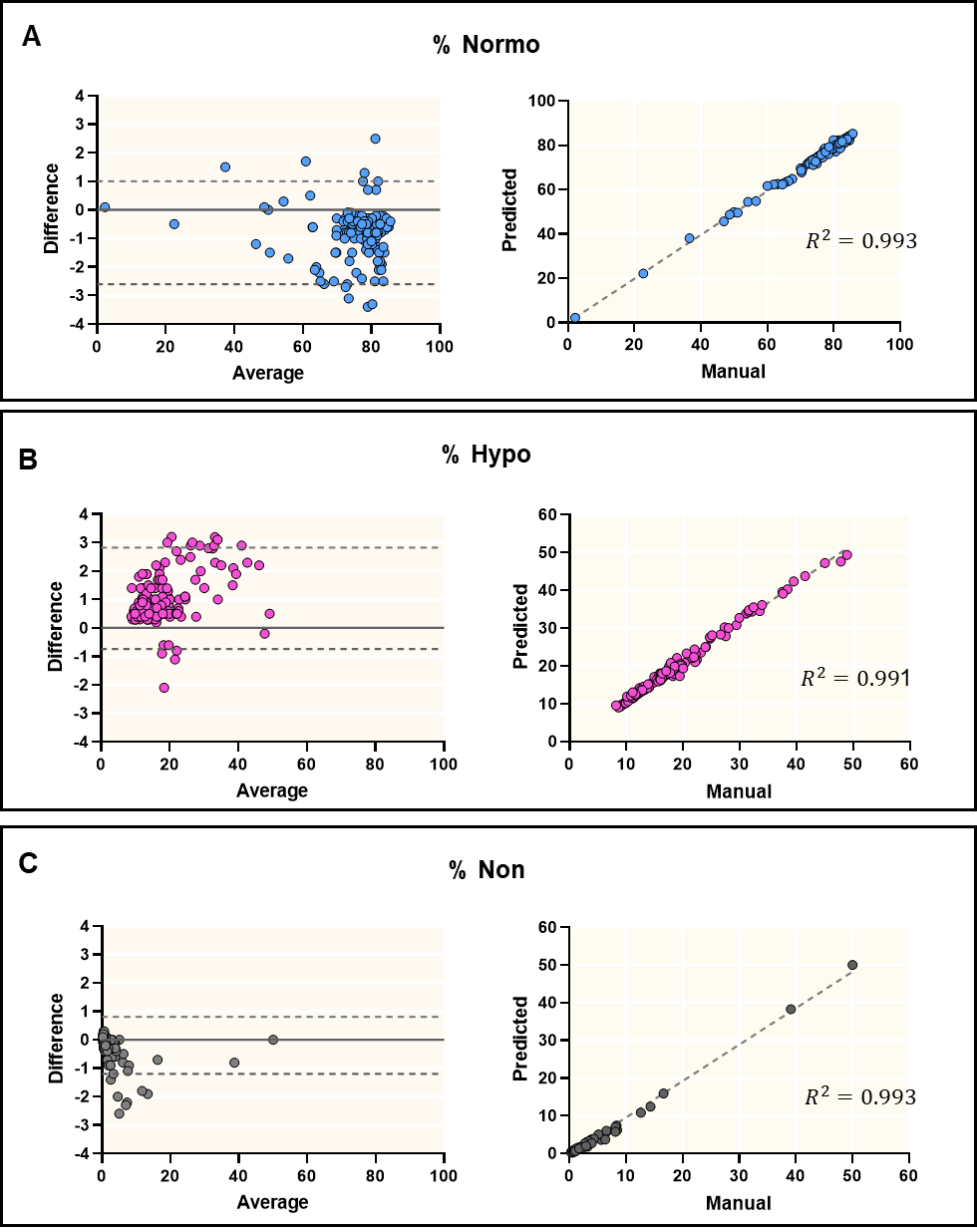


**Figure S4. Validation of the DL-based model for the automated segmentation**

Bland-Altman and Pearson's correlation plots of the aeration compartment retrieved either by manual segmentation or by implementing the DL model for the automated segmentation of BLM and BLM+NINT treated mice. (A) The Normo-aerated compartment expressed as percentage (%Normo), (B) the Hypo-aerated compartment expressed as percentage (%Hypo), (C) the Non-aerated compartment expressed as percentage (%Non). The horizontal dotted lines in the Bland-Altman plots show the upper and lower 95% limits of agreement (= bias ± 1.96 × SD). $R^{2}$: Squared Pearson’s correlation coefficient for the goodness of fit.

**Figure S5**

**E**

**%Gas_P01_**


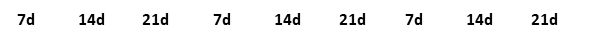

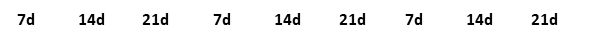

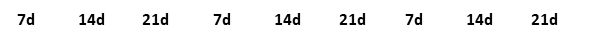

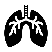


**Whole**


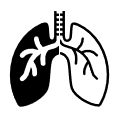

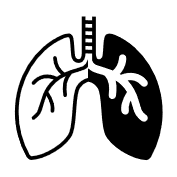


**Left**

**Right**


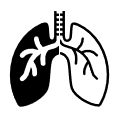

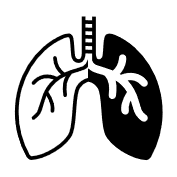


**A**

**B**

**C**

**D**

**SAL**

**Figure S5. Longitudinal assessment of some derived** $\boldsymbol{\mu}$**CT parameters measured from the whole lung and for separate left and right lungs in Saline mice.**

(A) Quantification of the Normo-aerated compartment expressed as a percentage (%Normo). (B) Quantification of the air content at the end of inspiration expressed as a percentage (%Gas_P01_). (C) Quantification of the Total Lung Volume at the end of expiration ($V_{P02}$). (D) Quantification of the Tidal Volume (TV). (E) Quantification of the non-gas volume (Tissue). Data were expressed as mean ± SEM. Statistical significance of differences between whole vs. left or right lungs was calculated by Two-way ANOVA followed by Dunnett’s t post-hoc test (* p < 0.05; ** p < 0.01; *** p < 0.001 vs. Whole lung).

**Table S1.** p-values derived from a paired Student’s t-test analysis comparing the biomarkers measured in the right and left lungs of SAL animals at each time-point.

| **Paired t-test: left and right lobes** | | | |
| --- | --- | --- | --- |
|  | Day 7 | Day 14 | Day 21 |
| %Normo | ns | ns | ns |
| %Gas_P01_ | ns | ns | ns |
| $V_{P02}$ | *** | *** | *** |
| TV | *** | *** | *** |
| Tissue | *** | *** | *** |

ns p>0.05; * p < 0.05; ** p < 0.01; *** p < 0.001
