# Supplementary material for: A fully automated micro‑CT deep learning approach for precision preclinical investigation of lung fibrosis progression and response to therapy
Source: Respir Res. 2023 May 9;24:126. doi: 10.1186/s12931-023-02432-3 (PMC10170869; doi:10.1186/s12931-023-02432-3)
Supplement: Supplementary file 2 — Additional file 2: Table S2. Data partitioning by time point, acquisition phase, and disease prevalence. Table S3. A summary of dice score main statistics in multi-view aggregation stage. [file 12931_2023_2432_MOESM2_ESM.docx]

**Supplementary Materials & Methods**

An early version of the DL model for automated segmentation was implemented by Vincenzi et al. [1].

A first multiclass 2D U-Net trained on subsampled µCT axial slices (down-sample factor = 4) in raw grey levels was used to extract a preliminary segmentation of lungs, airway, and heart from µCT scans and perform pre-processing and extract a bounding box in which the lung volume is entirely contained to identify the Region of Interest (ROI) in the higher resolution image. The ROI identified and converted to HU is then processed by three different 2D U-Nets trained separately in the axial, sagittal and coronal planes, obtained by extracting 2D slices along the x, y and z axes of the higher-resolution µCT scan. The predictions provided by the three U-Net planes are then combined to provide a final spatially coherent segmentation of the left and right lungs.

The final segmentation of whole lung and of the two separated lobes are used to automatically extracts the volumes ($V_{P01}$ and $V_{P02}$) and mean lung attenuations (MLA_P01_ and MLA_P02_) densities (measured in Hounsfield Units, HU).

Finally, whole, left and right segmentations are compartmentalized based on the corresponding voxel value in µCT into Normo-aerated ([–860, -435] HU), Hypo-aerated ((-435, -121) HU), non-aerated ([-121, +121] HU) and Hyper-inflated [-1040, -860] HU) compartments, according to the ‘HU preclinical ranges’ introduced by Mecozzi et al. [2]. All parameters (aeration degrees, FRC, etc..) involved in densitometric analysis are then computed.

The models employed in the proposed pipeline were trained using only µCTs from the BLM-induced lung fibrosis model in female mice. Such scans were divided into two datasets. Firstly, 167 µCTs (Table S2, *Dataset A*) allowed end-to-end learning of lung segmentation, then, the decoding path of those models was re-trained on 52 µCTs (Table S2, *Dataset B1*), allowing learning of right and left lung segmentation by transfer learning.

In this work, we enriched the original training dataset with a total of 184 scans related to the male murine model to increase the general capability of the densitometry pipeline. Specifically, µCTs from 46 subjects at two different time points (day 7 and day 21) at both the P01 and P02 respiratory phases were added, and the decoding path of the models trained on *Dataset A* was re-trained on 144 P01 and 112 P02 µCTs (Table S2, *Dataset B2*).

Table S2 shows data partitioning by time point, acquisition phase (end-expiration/end-inspiration), and disease prevalence.

Table S2. Data partitioning by time point, acquisition phase (end-expiration P02/end-inspiration P01), and disease prevalence

| DATASET SUMMARY | | | | | | | | | | | | |
| --- | --- | --- | --- | --- | --- | --- | --- | --- | --- | --- | --- | --- |
|  | **0 days** | | **7 days** | | **14 days** | | **21 days** | | **# Scans** | | **Prevalence** | |
|  | *P02* | *P01* | *P02* | *P01* | *P02* | *P01* | *P02* | *P01* | *P02* | *P01* | *P02* | *P01* |
| *Dataset A* | 4 | 5 | 41 | 15 | 35 | 21 | 87 | 26 | 167 | 67 | 84% | 92% |
| *Dataset B1* | 5 | 5 | 21 | 5 | 5 | 5 | 21 | 5 | 52 | 20 | 69% | 60% |
| *Dataset B2* | 5 | 5 | 67 | 51 | 5 | 5 | 67 | 51 | 144 | 112 | 96% | 94% |

Each µCT volume was segmented manually by trained operators by means of Analyze software (Analyze 12.0; Copyright 1986-2017, Biomedical Imaging Resource, Mayo Clinic, Rochester, MN)^[[1]](#footnote-1)^, according to Chiesi Farmaceutici S.p.A. well-established protocol.

µCT acquisitions and corresponding segmentations have been divided into three sets (training set, validation set, test set) ensuring that the µCTs acquired for each mouse at different time points were included in the same set. For each training, a 5-fold split approach was adopted to evaluate the prediction on each µCT in the dataset and the final model was trained afterwards. Training set and test set were respectively exploited to train and evaluate the network, whereas validation set was used both to prevent overfitting during the training process by early stopping regularization and to define the threshold needed to binarize the probability maps provided by the networks. Specifically, 5 different models were initially trained on 5 different splits of the dataset. A final model was then trained by setting the number of epochs and the binarization thresholds as medians of the parameters obtained in the splits to train the model on as many data as possible. Approximately 5% of the data was used as a control test set.

The models described in Vincenzi et al. [1] were developed in two stages. Initially, the k-fold-splitting procedure described using Dataset A was adopted to allow end-to-end learning of lung segmentation. Subsequently, the final single-view models obtained were used as a starting point to allow learning of right and left lung segmentation by transfer learning from models that segmented whole lung jointly. Specifically, the decoding path of the single-view models was retrained on the µCT and related Dataset B1 segmentation maps. This second step was also performed by adopting the same k-fold-splitting procedure.

In the approach described in Vincenzi et al. [1] only P02 scans were included in the training process by k-fold-splitting, while P01 scans were used as an additional test of the final models. In this work, we also included P01 scans in the training stage.

Table S3 shows the number of training, validation, and test sets for each step.

Table S3. Data Splitting in training, validation and test set for Dataset B2 in each split and in final training

| DATA SPLITTING | | | |
| --- | --- | --- | --- |
|  | **# train** | **# validation** | **#test** |
| *Dataset* | *B2* | *B2* | *B2* |
| *Split 1* | 180 | 24 | 52 |
| *Split 2* | 180 | 24 | 52 |
| *Split 3* | 182 | 22 | 52 |
| *Split 4* | 182 | 24 | 50 |
| *Split 5* | 184 | 22 | 50 |
| *Final* | 242 | 0 | 14 |

For model trainings, categorical cross-entropy was used as a loss function in single-view models. The Adam optimizer with learning rate = 0.0001 was adopted to optimize the network parameters. In each iteration, a mini-batch containing 4 slices randomly sampled from the training set was provided to the single view U-Net. The training process was stopped using early stopping criteria, with patience set to 10 epochs.

A data augmentation procedure was adopted: images and masks in the mini batch were modified on the fly during the training process with random rotations, shifts, and zoom factors to augment training and validation sets. The transformation parameters were extracted randomly from a uniform distribution range of maximum variation of [-5°,5°] for rotation, [-5%, +5%] for shifting and [-15%, +15%] for zooming.

The segmentations obtained with the proposed pipeline are compared to manual segmentations using the Dice Score (DSC) (Table S3). Specifically, DSC was used to evaluate the performance of the network as an overlap measure between the predicted and the manual segmentations. The Dice coefficient between two binary segmentations is defined as follows:

$$DSC=\frac{2\left| Manu\cap Pred \right|}{\left| Manu \right|+\left| Pred \right|}$$

where $Manu$ is the manual segmented volume and $Pred$ is the automatically segmented volume.

Table S4. A summary of dice score main statistics in multi-view aggregation stage

| MULTI-VIEW AGGREGATION RESULTS | | | |
| --- | --- | --- | --- |
|  | **Dice Score Left** | **Dice Score Right** | **Dice Score Total** |
| *Average* | 0.985 | 0.976 | 0.978 |
| *Standard Deviation* | 0.005 | 0.010 | 0.012 |
| *Median* | 0.986 | 0.978 | 0.982 |
| *IQR* | 0.005 | 0.011 | 0.012 |
| *5° Percentile* | 0.973 | 0.957 | 0.953 |
| *95° Percentile* | 0.991 | 0.986 | 0.990 |

Bland-Altman plots and Pearson’s correlation coefficient were also computed to compare values of the aeration compartments retrieved manually and automatically in the whole lung (Figure S2). As shown in the Bland Altman plots, almost all analyzed data are within the confidence intervals highlighted by the dashed gray lines. Moreover, the trend lines drawn from the scatterplots of Figure S2 shown a significant Pearson’s correlation between the manual and automatic measures, with a R^2^>0.99 in all compartments.

**References**

1. Vincenzi E, Fantazzini A, Basso C, Barla A, Odone F, Leo L, et al. A fully automated deep learning pipeline for micro-CT-imaging-based densitometry of lung fibrosis murine models. Respir Res. 2022 Nov 11;23(1):308.

2. Mecozzi L, Mambrini M, Ruscitti F, Ferrini E, Ciccimarra R, Ravanetti F, et al. In-vivo lung fibrosis staging in a bleomycin-mouse model: a new micro-CT guided densitometric approach. Sci Rep. 2020;

1. <https://www.analyzedirect.com> [↑](#footnote-ref-1)
